# Association Between Tumor Mutation Profile and Clinical Outcomes Among Hispanic-Latino Patients With Metastatic Colorectal Cancer

**DOI:** 10.3389/fonc.2021.772225

**Published:** 2022-01-24

**Authors:** Alexander Philipovskiy, Reshad Ghafouri, Alok Kumar Dwivedi, Luis Alvarado, Richard McCallum, Felipe Maegawa, Ioannis T. Konstantinidis, Nawar Hakim, Scott Shurmur, Sanjay Awasthi, Sumit Gaur, Javier Corral

**Affiliations:** ^1^ Department of Internal Medicine, Division of Hematology-Oncology, Texas Tech University Health Sciences Center Lubbock, Lubbock, TX, United States; ^2^ Department of Internal Medicine, Division of Hematology-Oncology, Texas Tech University Health Sciences Center El Paso, El Paso, TX, United States; ^3^ Department of Molecular and Translational Medicine, Division of Biostatistics & Epidemiology, Paul L. Foster School of Medicine, Texas Tech University Health Sciences Center El Paso, El Paso, TX, United States; ^4^ Department of Internal Medicine, Division of Gastroenterology, Texas Tech University Health Sciences Center El Paso, El Paso, TX, United States; ^5^ Department of Surgery, Southern Arizona VA Health Care System, University of Arizona, Tucson, AZ, United States; ^6^ Department of Surgery, Texas Tech University Health Sciences Center El Paso, El Paso, TX, United States; ^7^ Department of Pathology, Texas Tech University Health Sciences Center El Paso, El Paso, TX, United States

**Keywords:** colon cancer, Hispanic-Latino patients, APC, TP53, KRAS, Gnas, NOTCH, tumor mutation profile

## Abstract

In the United States, CRC is the third most common type of cancer and the second leading cause of cancer-related death. Although the incidence of CRC among the Hispanic population has been declining, recently, a dramatic increase in CRC incidents among HL younger than 50 years of age has been reported. The incidence of early-onset CRC is more significant in HL population (45%) than in non-Hispanic Whites (27%) and African-Americans (15%). The reason for these racial disparities and the biology of CRC in the HL are not well understood. We performed this study to understand the biology of the disease in HL patients. We analyzed formalin-fixed paraffin-embedded tumor tissue samples from 52 HL patients with mCRC. We compared the results with individual patient clinical histories and outcomes. We identified commonly altered genes in HL patients (*APC, TP53, KRAS, GNAS*, and *NOTCH)*. Importantly, mutation frequencies in the *APC* gene were significantly higher among HL patients. The combination of mutations in the *APC, NOTCH*, and *KRAS* genes in the same tumors was associated with a higher risk of progression after first-line of chemotherapy and overall survival. Our data support the notion that the molecular drivers of CRC might be different in HL patients.

## Introduction

According to the World Health Organization GLOBOCAN database, in 2019, approximately 1.8 million new colorectal cancer cases were diagnosed, and almost 861,000 deaths were reported. Colorectal cancer is the third most frequent type of cancer and the second leading cause of cancer-related death in the United States. According to the American Cancer Society, in 2021, approximately 147,950 individuals in the United States will be diagnosed with CRC and 53,500 will die from the disease. Notably, approximately 17,930 new cases of colorectal cancer (CRC) and 3640 deaths will occur in 2021 in individuals aged younger than 50 years (1). In the United States, the lifetime risk of CRC is approximately 6%, and the average age at diagnosis is 66 years. Unfortunately, approximately 40% of all patients with CRC have metastatic disease at initial presentation (2). Metastatic CRC carries a dismal prognosis, with a five-year survival rate of approximately 15%. Importantly, for a very select population with so-called oligometastatic disease, typically located in the liver, they can be cured after surgical resection (3). More than two decades ago, the introduction of oxaliplatin and irinotecan changed the paradigm for managing these patients. Combined chemotherapy with 5-fluorouracil (5-FU) and oxaliplatin (FOLFOX) or irinotecan (FOLFIRI) continues to be the best available frontline treatment for metastatic CRC, with an objective response rate of approximately 50% (4, 5). In addition, the recently introduced anti-epidermal growth factor receptor antibody (EGFR) and anti-vascular endothelial growth factor A antibody (VEGF) in combination with conventional chemotherapy have demonstrated significant improvements in clinical outcomes (6). Unfortunately, once the disease progresses, the outcome is dismal, with very few salvageable options available (7–9). Although we have learned that specific genes related to the DNA damage repair system, such as TGF-β1, thymidylate synthase, and kallikrein-related peptidase, are related to resistance to 5-FU and oxaliplatin, and that mutations in RAS family genes are responsible for resistance to anti-EGFR therapy, the general nature of resistance to chemotherapy is unknown.

No exact etiology for CRC has been identified. Some theories suggest the possible role of diet, lifestyle, environmental factors, inherited mutations, and racial disparity (10–13). Inherited CRCs can be attributed to hereditary non-polyposis CRC (HNPCC), familial adenomatous polyposis (FAP), and closely related variant syndromes. However, only 15%–30% of patients with newly diagnosed CRC fall into this category. The majority of CRC cases are sporadic (70%–85%) and have more biological variables than hereditary CRC cases (14).

A growing body of evidence suggests a significant role of racial disparity in the biology of CRC. For many decades, researchers have been moving toward a better understanding of the factors that contribute to colorectal cancer (CRC) health disparities (11). Patients race/ethnicity has been reported in multiple studies in the past as an important prognostic and predictive factor for clinical outcomes (9). Significant progress has been made toward a better understanding of the molecular landscape of colorectal cancer in general. The largest publicly available database, TCGA, provides a comprehensive molecular characterization of tumors from the colon and rectum. It is, however, important to emphasize that almost all public genomic databases have a limited representation of patients from “minority” ethnic/racial groups. In particular, in the TCGA database, only 3% of the genomic data have been collected from Hispanic patients (15). Therefore, it is crucial to characterize tumor mutation profiles among the HL population to better understand the biology of cancer among these patients, as well as the cancer-related outcome disparities observed in this group of patients.

In the present study, we aimed to characterize the mutational profile of mCRC in the HL population and its association with clinical outcomes.

## Materials and Methods

### Patient Population

The study protocol was reviewed and approved by the Institutional Review Board of the Texas Tech University Health Sciences Center El Paso (TTUHSCEP) before the commencement of the study. Due to the retrospective nature of the study, written informed consent was not required. All data/tissue samples were fully anonymized. In this study, we retrospectively reviewed the TTUHSCEP clinical databases from January 2011 to January 2021 to identify HL patients diagnosed with unresectable Stage III and IV CRC. Any patients with incomplete data on outcomes such as progression-free survival (PFS) were excluded from the study. We defined the primary tumor site into two categories: Right-sided (from the cecum to the transverse colon) and left-sided (from the spleen flexure to the rectum).

### Pathologic Assessment

Pathological diagnosis was determined during the initial evaluation. Standard immunohistochemical (IHC) staining was used. Hematoxylin and Eosin staining slides were reviewed by pathologists to select the area with the most abundant tumor tissues.

### Tumor Genome Sequencing

For this study, we retrospectively collected and analyzed the genome sequencing data (Foundation Medicine, Foundationone CDX Cambridge, MA, USA) of 52 patients with mCRC who were treated at the TTUHSCEP.

Briefly, patient DNA was extracted from FFPE samples. The assay employed a single DNA extraction method from routine FFPE biopsy or surgical resection specimens, 50–1000 ng of which underwent whole genome shotgun library construction and hybridization-based capture of all coding exons from 309 cancer-related genes, one promoter region, one noncoding (ncRNA), and selected intronic regions from 34 commonly rearranged genes, 21 of which also included coding exons. In total, the assay detected alterations in 324 genes. Using Illumina^®^ HiSeq 4000 (Illumina, Inc. San Diego, CA, USA) platform-hybrid capture, selected libraries were sequenced to high uniform depth (targeting >500× median coverage with >99% of exons at >100× coverage). Sequence data were then processed using a customized analysis pipeline designed to detect all classes of genomic alterations, including base substitutions, indels, copy number alterations (amplifications and homozygous gene deletions), and selected genomic rearrangements (for details go to: https://www.accessdata.fda.gov/cdrh_docs/pdf17/P170019S017B.pdf).

### Comparing our Data With International Databases

The frequency of cancer gene mutations discovered in our study was compared with previously published databases TCGA (The Cancer Genome Atlas) and data from some other national and international databases (16–19).

### Outcome Measures and Statistical Analysis

The primary clinical outcomes were progression after the first line of chemotherapy (yes vs. no) and overall survival (yes vs. no) at the end of the study. Age was described using mean and standard deviation (SD) while the rest all the considered categorical variables were presented using frequencies and percentages. The clinical-pathological characteristics including a number of chemotherapy cycles were compared between genders using Fisher’s exact test except for the age which was compared using an unpaired t-test. The proportion of each mutated gene in our patient cohort was compared with the corresponding proportion in other databases using Fisher’s exact tests. A binary variable cluster analysis was performed to identify the clustering among the mutated genes using hclustvar function under the R package ClustOfVar and the number of clusters was chosen based on the aggregation plot and the stability plot and accordingly three additional groups of mutated genes were created (a) individuals with presence or absence of mutations with *TP53* and *BRCA* (b) individuals with presence or absence of mutations with *GNAS* and *AURKA*, and (c) individuals with presence or absence of mutations with *APC*, *NOTCH*, and *KRAS*. The distributions of individual genes and combined genes based on variable cluster analyses were compared according to each outcome status (progression after the first line of chemotherapy and overall survival) using Fisher’s exact tests. P-values less than 5% were considered statistically significant. All statistical analyses were performed using STATA 17. We followed the statistical analysis and methods in biomedical research guidelines in this study (20).

## Results

From January 2011 to January 2021, we retrospectively analyzed the results of 52 patients with individual tumor gene mutation profiles. All patients had a pathologically confirmed diagnosis of adenocarcinoma of colon or rectum. The clinicopathological characteristics of the patients are shown in **Table 1**. In the study we observed 36 males (69.23%) and 16 females (30.77%). The average age of patients at diagnosis was 58.7 years. All patients identified themselves as Hispanic-Latinos (*n* = 52). The majority of patients had Stage IV disease (95%) at the time of diagnosis, and three patients progressed from Stage III to metastatic disease during the study period. All patients received at least one round of conventional chemotherapy (FOLFOX or FOLFIRI). Interestingly, 16 patients were stable after the completion of the first-line of chemotherapy for more than 12 months, and 11 remained stable after 36 months. After receiving first-line chemotherapy FOLFOX and being followed for eight years, one patient with metastatic disease remained stable. Thirty-six (69.23%) patients had left-sided primary tumors (the splenic flexure to the rectum), while 16 (30.76%) patients had right-sided tumors (the cecum to the transverse colon). The most common primary metastatic site was the liver (*n* = 40, 76.92%), followed by the lung (*n* = 5, 9.61%), the peritoneum (*n* = 3, 5.76%), and other sites (*n* = 4, 7.69%). The left-sided colon was the most prevalent location of primary tumors in males (*n* = 27, 75%); however, the distribution was more equal between sides (56.25% in the left-sided colon and 43.75% in the right-sided colon) in females (**Table 1**).

**Table 1:** Clinicopathological characteristics of patients in the entire cohort and by gender.

Clinicopathological characteristics	Overall	Female	Male	*p*-Value
	*N* = 52	16	36	
Age at diagnosis	58.67	57.06	59.39	0.47
(SD)	(10.64)	(14.7)	(8.41)	
Laterality of the primary tumor				0.21
Left-sided	36	9 (25%)	27 (75%)	
Right-sided	16	7 (43.75)	9 (56.25%)	
Metastasis				
Liver	40	12	28	
Lung	5	0	5	
Peritoneum	3	2	1	
Other	4	2	2	
Clinical stage				
IIIc/IV	1	1	0	
IV	51	15	36	

### Data Comparison With Other Cancer Databases

To demonstrate the diversity of the tumor gene expression profiles, we further compared our data to more extensive international cancer databases, such as TCGA (16–19, 21, 22) (which has a predominantly NHW population and a small portion of AA and HL patients) and one recently published study from China (19). The most commonly mutated genes were *APC* (92.3%), *TP53* (75%), *KRAS* (50%), *GNAS* (31%), *PICK3CA* (27%), and *NOTCH* (23%) (**Table 2**). Compared to other studies, mutation frequencies in the *APC* gene were significantly higher among HL patients with mCRC. The frequency of other common mutations did not reach a statistically significant value (**Table 2**).

**Table 2:** Prevalence of mutated genes in Hispanic patients with metastatic colorectal cancer in our study cohort compared to other databases.

Gene	TTUHSC	TCGA (Firehose)	TCGA (Nature 2012)	Pan Cancer Atlas	Chinese study (19)
	(M1)	(M1) (18)	(Mx) (16)	(Mx) (17)	(M0+M1)
	*n* = 52	*n* = 28	*n* = 224	*n* = 478	*n* = 630
	%	% (*p*-value)	% (*p*-value)	% (*p*-value)	% (*p*-value)
APC	92	75 (0.044)	75 (0.005)	73 (0.001)	71 (0.001)
TP53	75	57 (0.131)	54 (0.008)	62 (0.070)	77(0.734)
KRAS	50	36 (0.247)	42 (0.352)	40 (0.182)	50 (1.00)
GNAS	31	11 (0.056)	6 (<0.001)	4 (<0.001)	N/D
PIK3CA	27	N/D	20 (0.347)	26 (0.869)	18 (0.136)
NOTCH	23	11 (0.236)	15 (0.214)	16 (0.237)	N/D
ATM	21	N/D	11.2 (0.067)	11 (0.043)	N/D
FLT	21	N/D	5 (0.001)	11 (0.043)	N/D
SRC	15	N/D	N/D	N/D	N/D
MLL2	14	N/D	N/D	N/D	N/D

N/D, not determined; p-values were determined using Fisher’s exact tests. M0 – non-metastatic disease, M1 – metastatic disease, Mx - metastasis statuses not reported.

### Driver Gene Analysis

We examined the most frequently mutated genes to identify the potential genes of interest in our study cohort. The most frequently mutated genes were *APC* (92.3%), *TP53* (75%), *KRAS* (50%), *PICK3CA* (27%), and *NOTCH* (23%), which have been connected with colorectal cancer in a previous study, and contribute to cancer growth and development. Among the less commonly reported genes, we identified mutations in *GNAS* (31%), *ATM* (21%), and *MLL1* (4%) (**Table 2**).

**Figure 1:**
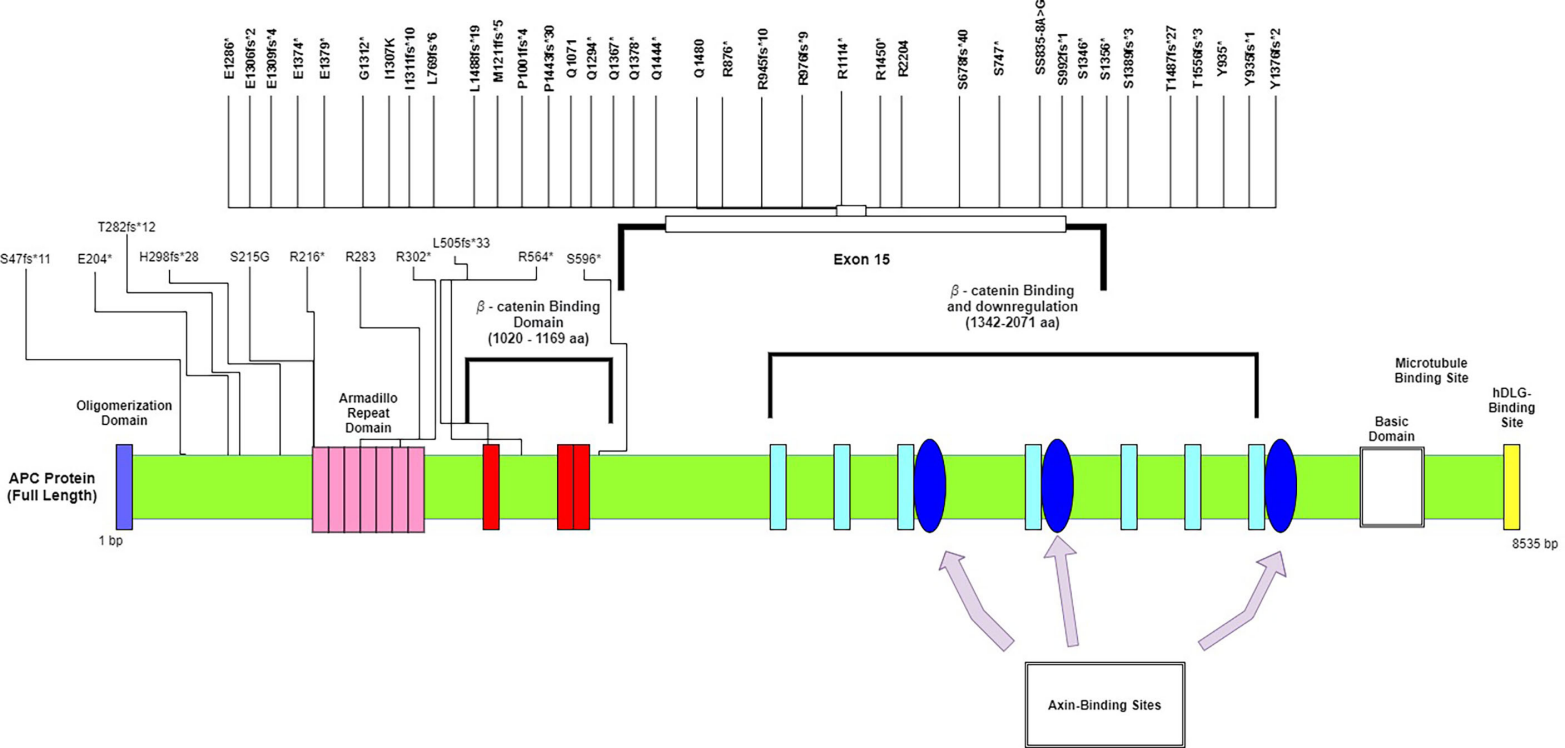
Mutational spectrum of APC in mCRC. The figure showed protein domains and the positions of specific mutations.

We did not see any significant differences in the distribution of *APC* mutations among different age groups or between left- and right-sided primary tumor sites. Compared to other studies, a significantly lower frequency of *APC* mutations was reported in the TCGA database (75%) and a recently published study from China (71%) (23) (**Table 2**). The majority of the mutations in *APC* (**Figure 1**), were frameshift mutations at 59.2%, followed by point mutations at 40.8%. The majority of point mutations (66%) were distributed between Codons 1200 and 1500, an area which has been previously described as a MCR (mutation cluster region) in the *APC* gene (24). In the MCR area, there are two hotspots for somatic mutations: Codons 1450 and 1309. We subsequently identified four tumors with mutations in Codon 1450 and two in Codon 1309. Mutations in the MCR region resulted in a truncated APC protein. *APC* mutations were more common in Hispanic males (97.22%) and less common among Hispanic females (81.25%, *p* = 0.081) (**Table 3**). An intriguing discovery was made when we used a variable cluster analysis for categorical variables to identify the group of genes related to outcomes. We found that in patients whose tumors had a combination of mutations in *APC, NOTCH*, and *KRAS* genes, the overall survival was much shorter (HR 4.39, 95%CI 1.09-17.74) and the risk of progression after first-line chemotherapy with mFOLFOX6 was increased (**Table 4**).

**Table 3:** Comparison of mutated genes, treatments, and progression between genders.

Factor	Gender		*p*-Value
	Female	Male	
N	16	36	
Number of Chemotherapy Cycles			0.41
1	5 (31.25%)	9 (25.00%)	
2	7 (43.75%)	11 (30.56%)	
>3	4 (25.00%)	16 (44.44%)	
TP53			0.73
0	3 (18.75%)	10 (27.78%)	
1	13 (81.25%)	26 (72.22%)	
PIK3CA			0.74
0	11 (68.75%)	27 (75.00%)	
1	5 (31.25%)	9 (25.00%)	
APC			0.081
0	3 (18.75%)	1 (2.78%)	
1	13 (81.25%)	35 (97.22%)	
KRAS			0.13
0	5 (31.25%)	21 (58.33%)	
1	11 (68.75%)	15 (41.67%)	
BRCA			0.51
0	13 (81.25%)	25 (69.44%)	
1	3 (18.75%)	11 (30.56%)	
NOTCH			0.3
0	14 (87.50%)	26 (72.22%)	
1	2 (12.50%)	10 (27.78%)	
GNAS			1.00
0	11 (68.75%)	25 (69.44%)	
1	5 (31.25%)	11 (30.56%)	
FLT			1.00
0	13 (81.25%)	28 (77.78%)	
1	3 (18.75%)	8 (22.22%)	
SRC			1.00
0	14 (87.50%)	30 (83.33%)	
1	2 (12.50%)	6 (16.67%)	
MLL2			1.00
0	14 (87.50%)	31 (86.11%)	
1	2 (12.50%)	5 (13.89%)	
Progression after first-line of chemotherapy			0.74
	11 (68.75%)	27 (75.00%)	

**Table 4:** Associations of genes and combinations of genes with disease progression and overall survival.

Genes	Progression after the first-line of chemotherapy		*p*-Value	Overall Survival		*p*-Value
	No	Yes		No	Yes	
NOTCH			0.86			0.094
No	11 (27.50%)	29 (72.50%)		35 (87.50%)	5 (12.50%)	
Yes	3 (25.00%)	9 (75.00%)		8 (66.67%)	4 (33.33%)	
SRC			0.062			0.16
No	14 (31.82%)	30 (68.18%)		35 (79.55%)	9 (20.45%)	
Yes	0 (0.00%)	8 (100%)		8 (100%)	0 (0.00%)	
AURKA			0.033			0.11
No	14 (33.33%)	28 (66.67%)		33 (78.57%)	9 (21.43%)	
Yes	0 (0.00%)	10 (100%)		10 (100%)	0 (0.00%)	
TP53 and BRCA			0.023			0.42
No	14 (34.15%)	27 (65.85%)		33 (80.49%)	8 (19.51%)	
Yes	0 (0.00%)	11 (100.00%)		10 (90.91%)	1 (9.09%)	
GNAS and AURKA			0.033			0.11
No	14 (33.33%)	28 (66.67%)		33 (78.57%)	9 (21.43%)	
Yes	0 (0.00%)	10 (100.00%)		10 (100.00%)	0 (0.00%)	
APC, NOTCH, and KRAS			0.55			
No	13 (28.26%)	33 (71.74%)		40 (86.96%)	6 (13.04%)	0.024 (HR 4.39, 95% CI 1.09-17.74
Yes	1 (16.67%)	5 (83.33%)		3 (50.00%)	3 (50.00%)	

**Figure 2:**
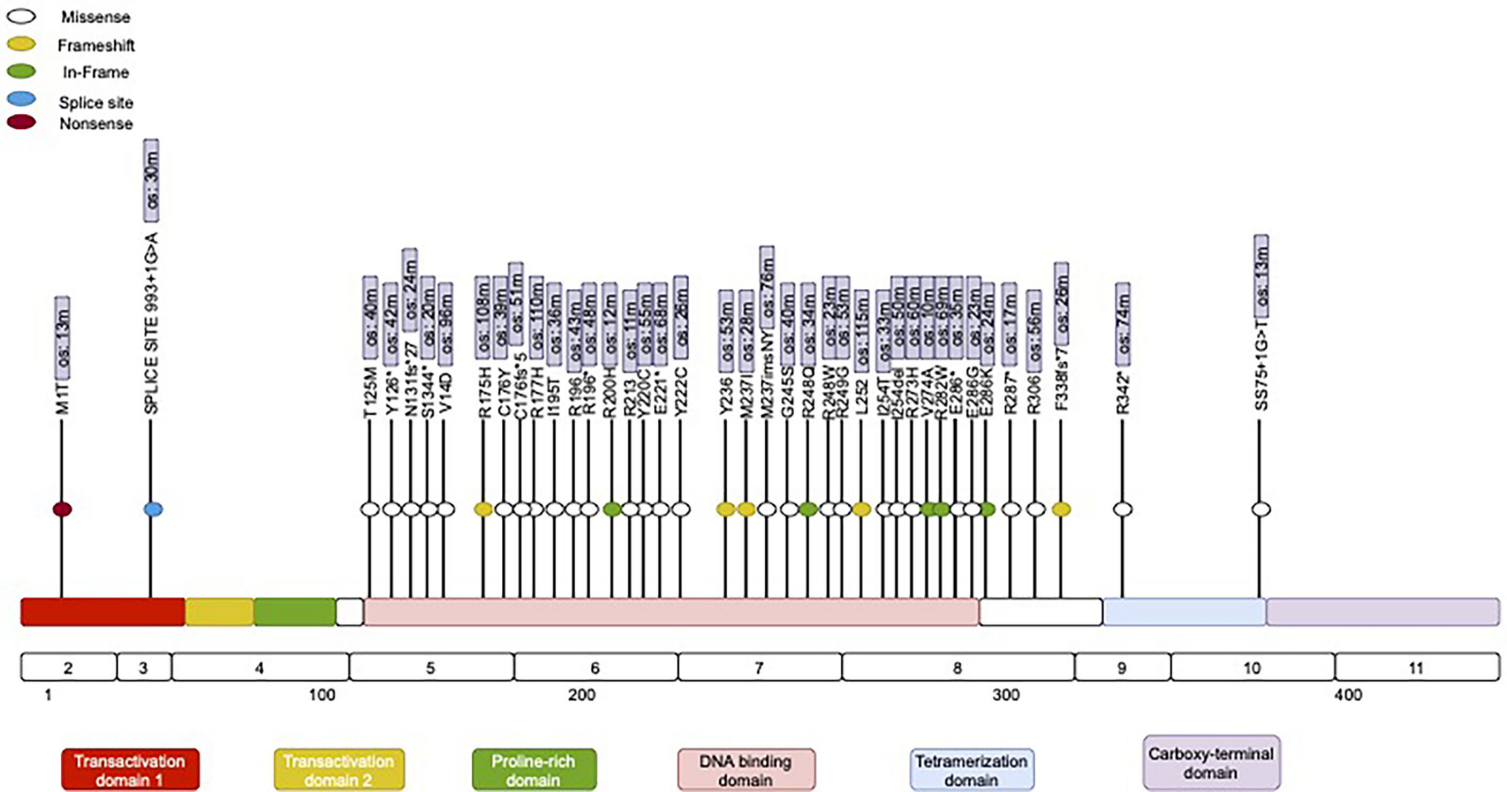
Mutational spectrum of TP53 in mCRC. The figure showed protein domains and the positions of specific mutations.

The majority of the mutations in the *TP53* gene (**Figure 2**) (86%), were missense mutations (single base substitution), followed by frameshift mutations in 11% and a complete loss of *TP53* in 3%. The majority of *TP53* mutations (92%) were distributed in the DBD (DNA-binding domain) area in clusters within Exons 5–8. Interestingly, previous data suggest a prognostic effect of *TP53* (25). In our study, however, we identified a trend for better outcomes for patients with *TP53* mutations in the tetrameric domain (Exons 8–10). We also identified the two most frequently mutated hotspots in *TP53*: Y220C and R196. Differences in the distribution of mutations in *TP53* between males and females, between left- and right-sides primary tumors, and among clinical outcomes were not statistically significant. Variable cluster analysis for categorical variables revealed that the presence of two mutations in *TP53* and *BRCA* (100% vs. 66%, *p* = 0.023) was associated with a higher incidence of recurrence after the first-line of chemotherapy with mFOLFOX6.

*KRAS* was the third most frequently mutated gene in our study population. We identified 26 cases with at least one *KRAS* gene mutation (**Table 3**). The most frequent mutations were in Codon 12 (61.5%), followed by Codon 13 (15.38%). Two cases had a *KRAS* gene amplification and double mutations in Codon 12. One case was detected with double mutations in the other two codons (G12S and Q61R). Mutations were less commonly seen in the *NRAS* gene (one case) and one in Codon 21 (21G). There were no significant differences in overall survival among patients with thouse mutations. We did not find statistically significant differences in the frequency of *KRAS* mutations among NHW, AA, and HL patients in the different colon cancer databases (**Table 2**). Interestingly, *KRAS* mutations were more common in Hispanic females (68.75%, 11 out of 16) and less common among Hispanic males (41.67% of 15 out of 36 patients), but because of the larger male population, the difference was not statistically significant (**Table 3**).

The majority of *GNAS* mutations detected in our analysis were classified as amplifications in 10 patients, and two patients had mutations in R201C and R201H, which have been connected with cancer progression in the past. The presence of mutations in both *GNAS* and *AURKA* in the same tumors (100% vs. 66.7%, *p* = 0.033) was associated with a significantly higher risk of recurrence after the first-line of chemotherapy (**Table 4**).

## Discussion

It has been demonstrated in the past that the age-adjusted incidence of colon cancer is approximately 43.8 per 100,000 patient population for HL, which is lower than in AA patients (45.7 per 100,000) but significantly higher than in non-Hispanic White (NHW) (38.6 per 100,000) patients (26, 27). Interestingly, the annual age-adjusted incidence of CRC has been declining over the last two decades in the HL population. Recently, however, new epidemiological data have suggested that despite the overall declining incidence of new CRC cases among HL patients, the onset of the disease occurs approximately 10 years earlier in HL patients than the average age reported for the NHW and AA populations (28). In addition, there has been a 45% increase in CRC incidence among Hispanic patients aged 20–49 years (29). It is unclear whether there are underlying biological and genetic drivers of colon cancer that are more prevalent in HL patients (30). Specifically, there is a gap in our knowledge regarding the genetic mutation profiles of different racial/ethnic subgroups, because only a few studies have addressed genetic diversity in HL patients, especially those with mCRC (30, 31).

Colorectal cancer is a heterogeneous disease that presents with different clinical features, responses to chemotherapy, and patient outcomes. At present, there is no consensus on the classification of colon cancer. The commonly referred to classifications in the literature subdivide colon cancer into four distinct consensus molecular subtypes (CMS) based on the tumor gene expression profiles (32). These subtypes are characterized by different genomic profiles, responses to chemotherapy, and subsequent clinical outcomes (33). Briefly, the first subtype is CMS-1-MSI-immune, which is found in approximately 14% of patients and is characterized by hypermutations due to defective DNA mismatch repair with microsatellite instability and MLH1 silencing (34, 35). The clinical CMS-1 subtype is connected to favorable outcomes, but only in patients with early-stage disease. CMS-2 accounts for 39% of all subtypes and is characterized by an indolent progression course with favorable five-year survival rates of 77% (36). The gene expression profile of CMS-2 predominantly displays epithelial signatures with prominent Wnt and Myc signaling activation pathways, and often reveals a loss of tumor suppressor genes (*APC* and *TP53*) and gains of oncogenes (*KRAS* and *PIK3CA*). CMS-3 represents 13% of all cases and is also characterized by a favorable five-year survival rate of 75%. It typically has genomic features consistent with chromosomal instability and has the highest rate of *KRAS* alterations among all subtypes (37). The CMS-4 subtype represents 23% of cases and is characterized by worse clinical outcomes. The gene expression profiles display a mesenchymal phenotype that is considered to be proinflammatory, has a high number of the TGFβ signaling characteristics of carcinoma-associated fibroblasts, displays angiogenesis, and has an inflammatory microenvironment with prominent innate immune cells (36, 38). The most commonly mutated genes are *APC, KRAS*, and *PIK3CA* (32). Because we did not analyze the individual tumor gene expression profiles in our study, it was impossible to precisely determine the distribution of the abovementioned subgroups among our patients. However, in our study, the most prevalent mutated genes were *APC* (92%), *TP5*3 (75%), and *KRAS* (50%). The combination of mutations in *APC, TP53*, and *KRAS* was detected in 15 (28.8%) patients. Interestingly, 2 of 15 patients had a short survival period (12 months) and constant progression, despite multiple rounds of chemotherapy. In our opinion, based on their clinical data and tumor mutation profiles, they might represent the CMS-4 subtype of CRC. The other 13 patients probably had the CMS-2 subtype, which clinically behaves less aggressively.

The most commonly altered gene in the HL population in our study was *APC* (92%) (**Figure 1**). It has been demonstrated in the past that most sporadic CRCs have *APC* somatic mutations (70%–80%), and the most commonly mutated area appears to be between Codons 1300 and 1500 (the MCR region) (13, 39). The *APC* gene is located on chromosome 5q21–q22, is composed of 8535 nucleotides, and encodes a 310 kDa protein. Approximately 75% of the coding sequence is located on Exon 15, which is reportedly the most common region for both germline and somatic mutations. Since the discovery of the *APC* gene in 1992, it has been shown to be involved in many signaling pathways and to play a critical role in colorectal tumorigenesis (40). It is well known as a tumor suppressor that is a negative regulator of the Wnt/b-catenin signaling pathway. Alterations in the *APC* gene lead to the expression of truncated protein products, resulting in activation of the Wnt signaling pathway and deregulation of many other cellular processes. Because APC is a multidomain protein and serves multiple functions through binding with different partners, it is possible—and some data suggest—that some form of C-terminally truncated APC proteins may have gain-of-function properties beyond the well-established loss of tumor-suppressive function (41). It has been demonstrated that the Wnt pathway interacts with MAPK, and dysregulation in one pathway may enhance MAPK activity in the other (42). It has been experimentally proven that activation of the Wnt signaling pathway is mediated by secreted WNT ligands binding to LRP5/6 receptors and the frizzled receptor FZD, which induces recruitment of the protein destruction complex to LRP receptors and subsequent phosphorylation of the Ser/Pro-rich motif of the LRP cytoplasmic domain *via* GSK3. The secretion of WNT ligands mainly depends on acylation by Porcupine (PORCN). PORCN is a membrane-bound O-acyltransferase that mediates the palmitoylation of WNT ligands to induce their secretion. Although the reactivation of altered APC has been accomplished *in vitro* and has demonstrated complete tumor regression (43, 44), it has never been validated in clinical trials. The best approach currently in use is to suppress an activated Wnt pathway by inhibiting the secretion of WNT ligands. Thus, multiple PORCN and FZD inhibitors and monoclonal antibodies against FZD receptors have been tested in clinical trials for a variety of solid tumors. The data from these studies are not mature at this point; however, we have learned more about the toxicity of PORCN and FZD inhibitors. Research has shown that the majority of these drugs cause severe GI symptoms and bone demineralization (45).

The second most commonly altered gene among HL patients in our study was *TP53* (75%) (**Figure 2**). *TP53* is widely considered to be a guardian of the genome because of its critical function in maintaining genome integrity, regulating the cell cycle, and initiating apoptosis (46). Multiple studies have reported that the frequency of *TP53* mutations in colorectal cancer ranges from 50% to 80%. Interestingly, not all of the published studies in the field have reported poor outcomes for patients with mutated *TP53* (47). For instance, there is no association between altered *TP53* and outcomes for patients with metastatic triple-negative breast cancer (48). In contrast, mutated *TP53* is connected with poor survival in patients with hormonal receptor-positive breast cancer and non-small cell lung cancer, but the exact mechanism behind *TP53* oncogenesis is unclear (49). Some researchers hypothesize that mutated p53 gains a novel function, known as a “tumor-transforming function,” which provides tumor cells an advantage in uncontrolled proliferation. The mutated p53 protein might serve as a negative inhibitor compared to wild-type p53 and thus may allow uncontrolled proliferation (50). Multiple compounds have been tested in clinical trials over the past few decades, with the aim of reactivating the mutated p53 protein or converting it to a wild-type protein; however, at present, this approach remains experimental and no approved treatment option is available to address *TP53* mutation or loss (51). More promising compounds such as AZD1775, APR-246, and COTI-2 have been found to exhibit anticancer activity in preclinical models (52).

Approximately 40% of CRCs have *RAS* mutations, and almost all of these mutations are located at Codons 12, 13, or 61 (53, 54). In our patient population, the *RAS* mutation was identified as the third most common mutation. We identified 26 cases with at least one *RAS* gene mutation. *KRAS* is a small GTPase (21 kDa) that binds guanosine triphosphate and diphosphate nucleotides. It is activated when bound to GTP and deactivated when bound to GDP. Mutations in Codons G12, G13, or Q61 commonly cause constitutive activation of KRAS. Activated KRAS binds and activates RAF family kinases (RAF1, BRAF, and ARAF), subsequently leading to uncontrolled proliferation and other processes causing cancer development and spread (55). KRAS can also regulate other signaling pathways, such as PI3K-AKT, PLC-PKC, and RAL, which are also known to be involved in cancer progression (56). For unclear reasons, mutations in *KRAS* are more common in pancreatic (80%–90%), colorectal (40%–60%), and lung cancers (30%); in contrast, mutations are rarely found in breast (1%–2%) and head and neck cancers (1%) (56–58). Controversy exists about the role of *KRAS* mutations as a clinical marker for patient outcomes. Some studies have suggested that *KRAS* mutation status might be used as a biomarker to predict responses to treatment and patient outcomes. For instance, *KRAS* mutations in patients with metastatic pancreatic cancer have been shown to be clinically correlated with poor responses to first-line chemotherapy (gemcitabine), with an objective response rate of only 11.3%, in contrast to 26.2% in patients with intact *KRAS* (59). A retrospective analysis of 273 patients with mCRC demonstrated a significant correlation between *KRAS* status and clinical outcomes. Some data have shown that patients with a mutated *KRAS* Codon 13 have more aggressive disease than those with mutations in Codon 12, but much controversy still exists (60, 61). Recent progress has been made in targeting *KRAS (G12C)* with a specific covalent inhibitor, such as ARS-853, AMG510, MRTX849, or ARS3248 (62). The FDA recently granted accelerated approval for the first-in-class KRAS (G12C) inhibitor sotorasib (Amgen Inc.) for patients with locally advanced or metastatic non-small cell lung cancer. This approval was granted on the basis of the objective response rate and the duration of the response demonstrated in the single-arm CodeBreaK100 trial. Another inhibitor, MRTX849, which was developed by Mirati Therapeutics, entered clinical trials in humans in January 2021 and has been used alone or in combination with the PD-1 inhibitor pembrolizumab or afatinib in patients with lung cancer or the anti-VGFR antibody cetuximab in patients with colon cancer (NCT03785249). ARS 3248 is another KRAS inhibitor developed by Johnson and Johnson and Wellspring Bioscience that entered clinical trials in humans in July 2019; however, for unclear reasons, the study was terminated (NCT04006301). Some data suggest the poor efficacy of the available KRAS inhibitors in colon cancer, and Genentech opened two clinical trials of the GDC-6036 compound in combination with one of the following compounds: atezolizumab (NSCLC) or cetuximab, bevacizumab (mCRC), or erlotinib (NSCLC). An alternative approach might be to target KRAS-upregulated pathways, such as PI3K-AKT, PLC-PKC, and RAL, in combination with KRAS inhibitors.

*GNAS* is a known oncogene that was first described in growth hormone-secreting pituitary adenomas and has been found to be mutated in some cancers. The most common mutation hotspot is at Codon 201 (63, 64). *GNAS* Codon 201 mutations are particularly frequent in intrapapillary mucinous neoplasia’s of the pancreas (65). The major product of the *GNAS* locus, the Gsα subunit of heterotrimeric G-proteins, acts to transduce signals from G-protein coupled receptors (GPCRs) to the effector enzyme adenylate cyclase in the G-stimulatory (Gs) pathway, leading to the production of cyclic AMP (cAMP). Both R201C and R201H mutations result in constitutive activation of Gsα and autonomous cAMP production (66). Interestingly, *GNAS* mutations are frequently accompanied by alterations in the *KRAS* gene (65). Activating mutations in *KRAS* and, to a lesser extent, its downstream effector *BRAF* are frequent events in colon cancer. Data from published full exome sequences of colorectal cancer have suggested that *GNAS* mutations are quite often accompanied by mutations in *KRAS* and/or *BRAF* (67). The amplification of GNAS has been shown to be connected with resistance to cetuximab in patients with metastatic colorectal cancer (RAS w/t tumor) and also to be associated with poor progression-free survival in patients with ovarian cancer (68, 69).

## Conclusions

This study represents one of the first studies of HL patients with mCRC focusing on distinctive genomic alterations. Compared to other studies, the mutation frequencies in the *APC* gene were significantly higher among HL patients with mCRC. Interestingly, this *APC* mutation was more common in Hispanic males (97.22%) and less common in Hispanic females 81.25% (*p* = 0.081), and was more likely to occur in the left colon. The combination of mutations in the *APC, NOTCH*, and *KRAS* genes in the same tumors was associated with a higher risk of progression after the first-line of chemotherapy and worse overall survival. In addition, combinations of two mutations in the same tumors, such as *TP53* with *BRCA* and *GNAS* with *AURKA*, were associated with a significantly higher risk of progression after the first round of chemotherapy.

Our study supports the genomic heterogeneity among NHW, AA, Asian, and HL individuals. If confirmed in larger trials, this could contribute to improvements in diagnostic and therapeutic approaches for these patients.

## Data Availability Statement

The datasets presented in this study can be found in online repositories. The names of the repository/repositories and accession number(s) can be found in the article/**Supplementary Material**.

## Ethics Statement

The study was approved by the Texas Tech University Institutional Review Board. Written informed consent for participation was not required for this study in accordance with the national legislation and the institutional requirements.

## Author Contributions

AP: Conceptualization, methodology and prepared the draft of the article. RG: data curation, visualization and editing of the article. AD: methodology, software, validation and review and editing of the article. LA: software, validation and review and editing of the article. G.R. methodology, and review and editing of the article. RM: methodology, and review and editing of the article. FM: methodology, and review and editing of the article. IK: methodology, and review and editing of the article. NH: methodology, and review and editing of the article. SS: methodology, and review and editing of the article. SA: methodology, and review and editing of the article. SG: methodology, and review and editing of the article. JC: methodology, and review and editing of the article. All authors read and agreed to the published version of the manuscript.

## Conflict of Interest

The authors declare that the research was conducted in the absence of any commercial or financial relationships that could be constructed as a potential conflict of interest.

## Publisher’s Note

All claims expressed in this article are solely those of the authors and do not necessarily represent those of their affiliated organizations, or those of the publisher, the editors and the reviewers. Any product that may be evaluated in this article, or claim that may be made by its manufacturer, is not guaranteed or endorsed by the publisher.
