# Supplementary material for: Association Between Tumor Mutation Profile and Clinical Outcomes Among Hispanic-Latino Patients With Metastatic Colorectal Cancer
Source: Front Oncol. 2022 Jan 24;11:772225. doi: 10.3389/fonc.2021.772225 (PMC8819001; doi:10.3389/fonc.2021.772225)
Supplement: Supplementary file 1 [file DataSheet_1.docx]

**Appendix 1**

**FoundationOne CDx-standard protocol**

<https://www.accessdata.fda.gov/cdrh_docs/pdf17/P170019S017B.pdf>

Sequencing is performed using off-board clustering on the Illumina cBot with patterned flow cell technology to generate monoclonal clusters from a single DNA template followed by sequencing using sequencing by synthesis (SBS) chemistry on the Illumina HiSeq 4000. Fluorescently labeled 3′-blocked dNTPs along with a polymerase are incorporated through the flow cell to create a growing nucleotide chain that is excited by a laser. A camera captures the emission color of the incorporated base and then is cleaved off. The terminator is then removed to allow the nucleotide to revert to its natural form and to allow the polymerase to add another base to the growing chain. A new pool of fluorescently labeled 3′-blocked dNTPs are added with each new sequencing cycle. The color changes for each new cycle as a new base is added to the growing chain. This method allows for millions of discrete clusters of clonal copies of DNA to be sequenced in parallel.

Sequence data are analyzed using proprietary software developed by FMI. Sequence data are mapped to the human genome (hg19) using Burrows-Wheeler Aligner (BWA) v0.5.9.4 PCR duplicate read removal and sequence metric collection are performed using Picard 1.47 (http://picard.sourceforge.net) and SAMtools 0.1.12a. Local alignment optimization is performed using Genome Analysis Toolkit (GATK) 1.0.4705. Variant calling is performed only in genomic regions targeted by the test. Base substitution detection is performed using a Bayesian methodology, which allows for the detection of novel somatic alterations at low mutant allele frequency (MAF) and increased sensitivity for alterations at hotspot sites through the incorporation of tissue-specific prior expectations. Reads with low mapping (mapping quality < 25) or base calling quality (base calls with quality ≤ 2) are discarded. Final calls are made at MAF ≥ 5% (MAF ≥ 1% at hotspots).

To detect indels, de novo local assembly in each targeted exon is performed using the de-Bruijn approach. Key steps are:

Collecting all read-pairs for which at least one read maps to the target region.

Decomposing each read into constituent k-mers and constructing an enumerable graph representation (de-Bruijn) of all candidate non-reference haplotypes present.

Evaluating the support of each alternate haplotype with respect to the raw read data to generate mutational candidates. All reads are compared to each of the candidate haplotypes via ungapped alignment, and a read ‘vote’ for each read is assigned to the candidate with best match. Ties between candidates are resolved by splitting the read vote, weighted by the number of reads already supporting each haplotype. This process is iterated until a ‘winning’ haplotype is selected.

Aligning candidates against the reference genome to report alteration calls.

Filtering of indel candidates is carried out similarly to base substitutions, with an empirically increased allele frequency threshold at repeats and adjacent sequence quality metrics as implemented in GATK: % of neighboring bases mismatches < 25%, average neighboring base quality > 25, average number of supporting read mismatches ≤ 2. Final calls are made at MAF ≥ 5% (MAF ≥ 3% at hotspots).
