# Supplementary material for: Association Between Tumor Mutation Profile and Clinical Outcomes Among Hispanic-Latino Patients With Metastatic Colorectal Cancer
Source: Front Oncol. 2022 Jan 24;11:772225. doi: 10.3389/fonc.2021.772225 (PMC8819001; doi:10.3389/fonc.2021.772225)
Supplement: Supplementary file 2 [file DataSheet_2.docx]

**Appendix 2**

**List of all mutated genes, reported by Foundation Medicine**

| **#** | **Gene** | **Cases** | **%** | **#** | **Gene** | **Cases** | **%** | **#** | **Gene** | **Case** | **%** |  |
| --- | --- | --- | --- | --- | --- | --- | --- | --- | --- | --- | --- | --- |
| **1** | **ATM** | 12 | 23.08 | **76** | **RICTOR** | 2 | 3.85 | **151** | **PARP2** | 2 | 3.85 |  |
| **2** | ***MUTYH** | 5 | 9.62 | **77** | **KMT2A (MLL)/A53V** | 4 | 7.69 | **152** | **WHSC1** | 2 | 3.85 |  |
| **3** | **CCND2** | 3 | 5.77 | **78** | **CBL** | 2 | 3.85 | **153** | **QKI** | 3 | 5.77 |  |
| **4** | **FBXW7** | 6 | 11.54 | **79** | **CDKN2A/B** | 2 | 3.85 | **154** | **MET** | 3 | 5.77 |  |
| **5** | **ARID1A** | 6 | 11.54 | **80** | **PDK1** | 1 | 1.92 | **155** | **MST1R** | 2 | 3.85 |  |
| **6** | **SDHA** | 1 | 1.92 | **81** | **FGF23** | 3 | 5.77 | **156** | **PTPN** | 2 | 3.85 |  |
| **7** | **CREBBP** | 7 | 13.46 | **82** | **JAK1** | 3 | 5.77 | **157** | **PIK3C2G** | 3 | 5.77 |  |
| **8** | **SMAD2** | 1 | 1.92 | **83** | **NOTCH** | 16 | 30.77 | **158** | **PRKCI** | 2 | 3.85 |  |
| **9** | **CYLD** | 1 | 1.92 | **84** | **HSD3B1** | 2 | 3.85 | **159** | **ACVR1B** | 2 | 3.85 |  |
| **10** | **CDK8** | 7 | 13.46 | **85** | **BRAF V600E** | 1 | 1.92 | **160** | **BAP1** | 2 | 3.85 |  |
| **11** | **CDH1** | 3 | 5.77 | **86** | **BRAF D594N** | 1 | 1.92 | **161** | **CSF3R** | 2 | 3.85 |  |
| **12** | **R295W** | 2 | 3.85 | **87** | **RB1** | 3 | 5.77 | **162** | **CTCF** | 4 | 7.69 |  |
| **13** | **KMT2A** | 11 | 21.15 | **88** | **IKZF1** | 3 | 5.77 | **163** | **RNF43** | 3 | 5.77 |  |
| **14** | **ARFRP1** | 6 | 11.54 | **89** | **PIK3C2B** | 2 | 3.85 | **164** | **HDAC1** | 2 | 3.85 |  |
| **15** | **MCL1** | 1 | 1.92 | **90** | **BCL2L1** | 9 | 17.31 |  |  |  |  | |
| **16** | **CDK8** | 7 | 13.46 | **91** | **MUTYH** | 4 | 7.69 |  |  |  |  |  |
| **17** | **BCL2L1** | 12 | 23.08 | **92** | **CD274** | 1 | 1.92 |  |  |  |  |  |
| **18** | **SMAD4** | 10 | 19.23 | **93** | **GATA4** | 2 | 3.85 |  |  |  |  |  |
| **19** | **SOX9** | 8 | 15.38 | **94** | **CDC73** | 2 | 3.85 |  |  |  |  |  |
| **20** | **MDM2** | 1 | 1.92 | **95** | **CDK12** | 2 | 3.85 |  |  |  |  |  |
| **21** | **ARID1A** | 6 | 11.54 | **96** | **NRAS** | 2 | 3.85 |  |  |  |  |  |
| **22** | **BRCA2** | 17 | 32.69 | **97** | **FAM123B** | 6 | 11.54 |  |  |  |  |  |
| **23** | **PAX5** | 1 | 1.92 | **98** | **ERBB3** | 3 | 5.77 |  |  |  |  |  |
| **24** | **FLT3** | 10 | 19.23 | **99** | **POLD1** | 4 | 7.69 |  |  |  |  |  |
| **25** | **ABL1** | 5 | 9.62 | **100** | **IGF1R** | 1 | 1.92 |  |  |  |  |  |
| **26** | **PALB2** | 4 | 7.69 | **101** | **MDM** | 2 | 3.85 |  |  |  |  |  |
| **27** | **DDR1** | 1 | 1.92 | **102** | **MAP3K1** | 5 | 9.62 |  |  |  |  |  |
| **28** | **KDR** | 4 | 7.69 | **103** | **SETD2** | 3 | 5.77 |  |  |  |  |  |
| **29** | **ASXL1** | 9 | 17.31 | **104** | **RNF43** | 3 | 5.77 |  |  |  |  |  |
| **30** | **ATRX** | 3 | 5.77 | **105** | **KDM5A** | 2 | 3.85 |  |  |  |  |  |
| **31** | **AURKA** | 11 | 21.15 | **106** | **PDGFRA** | 1 | 1.92 |  |  |  |  |  |
| **32** | **ALK** | 3 | 5.77 | **107** | **FGF** | 14 | 26.92 |  |  |  |  |  |
| **33** | **BRCA2** | 17 | 32.69 | **108** | **FLT3** | 10 | 19.23 |  |  |  |  |  |
| **34** | **ATRX** | 7 | 13.46 | **109** | **PALB2** | 4 | 7.69 |  |  |  |  |  |
| **35** | **KDR** | 4 | 7.69 | **110** | **RET** | 2 | 3.85 |  |  |  |  |  |
| **36** | **DDR1** | 1 | 1.92 | **111** | **FUBP1** | 2 | 3.85 |  |  |  |  |  |
| **37** | **PALB2** | 5 | 9.62 | **112** | **ZNF703** | 1 | 1.92 |  |  |  |  |  |
| **38** | **ARID1A** | 6 | 11.54 | **113** | **TBX3** | 2 | 3.85 |  |  |  |  |  |
| **39** | **SOX9** | 8 | 15.38 | **114** | **IKZF1** | 3 | 5.77 |  |  |  |  |  |
| **40** | **KEL** | 3 | 5.77 | **115** | **PIK3C2B** | 2 | 3.85 |  |  |  |  |  |
| **41** | **CDK12** | 2 | 3.85 | **116** | **CD274** | 1 | 1.92 |  |  |  |  |  |
| **42** | **EGFR** | 3 | 5.77 | **117** | **CDC73** | 2 | 3.85 |  |  |  |  |  |
| **43** | **SF3B1** | 1 | 1.92 | **118** | **NRAS** | 2 | 3.85 |  |  |  |  |  |
| **44** | **BCOR** | 7 | 13.46 | **119** | **CDK6** | 1 | 1.92 |  |  |  |  |  |
| **45** | **DNMT3A** | 2 | 3.85 | **120** | **CARD11** | 2 | 3.85 |  |  |  |  |  |
| **46** | **CTNNB1** | 2 | 3.85 | **121** | **HSD3B1** | 2 | 3.85 |  |  |  |  |  |
| **47** | **SRC** | 9 | 17.31 | **122** | **MAP3K1** | 3 | 5.77 |  |  |  |  |  |
| **48** | **EPHA3** | 3 | 5.77 | **123** | **MLL2** | 8 | 15.38 |  |  |  |  |  |
| **49** | **ARFRP1** | 6 | 11.54 | **124** | **NTRK2** | 2 | 3.85 |  |  |  |  |  |
| **50** | **ERBB4** | 4 | 7.69 | **125** | **FGF** | 8 | 15.38 |  |  |  |  |  |
| **51** | **KLHL6** | 1 | 1.92 | **126** | **ARID1A** | 1 | 1.92 |  |  |  |  |  |
| **52** | **EPHA3** | 3 | 5.77 | **127** | **AXL** | 4 | 7.69 |  |  |  |  |  |
| **53** | **SGK1** | 3 | 5.77 | **128** | **TSC1/2** | 13 | 25.00 |  |  |  |  |  |
| **54** | **ATR** | 7 | 13.46 | **129** | **ARAF** | 4 | 7.69 |  |  |  |  |  |
| **55** | **FLT1** | 9 | 17.31 | **130** | **BRD4** | 1 | 1.92 |  |  |  |  |  |
| **56** | **ARFRP1** | 6 | 11.54 | **131** | **CD22** | 1 | 1.92 |  |  |  |  |  |
| **57** | **FANCC** | 2 | 3.85 | **132** | **POLD1** | 3 | 5.77 |  |  |  |  |  |
| **58** | **DIS3** | 7 | 13.46 | **133** | **RAD51C** | 1 | 1.92 |  |  |  |  |  |
| **59** | **TNFAIP3** | 5 | 9.62 | **134** | **EPHB4** | 1 | 1.92 |  |  |  |  |  |
| **60** | **BCORL1** | 2 | 3.85 | **135** | **CDKN1B** | 1 | 1.92 |  |  |  |  |  |
| **61** | **SPEN** | 3 | 5.77 | **136** | **CUL4A** | 3 | 5.77 |  |  |  |  |  |
| **62** | **ARID1A** | 6 | 11.54 | **137** | **RAD21** | 5 | 9.62 |  |  |  |  |  |
| **63** | **VEGFA** | 1 | 1.92 | **138** | **MTOR** | 2 | 3.85 |  |  |  |  |  |
| **64** | **NF1** | 5 | 9.62 | **139** | **SUFU** | 3 | 5.77 |  |  |  |  |  |
| **65** | **FBXW7** | 6 | 11.54 | **140** | **POLE** | 3 | 5.77 |  |  |  |  |  |
| **66** | **ARFRP1** | 6 | 11.54 | **141** | **DOT1L** | 2 | 3.85 |  |  |  |  |  |
| **67** | **KMT2A** | 11 | 21.15 | **142** | **SMARCA4** | 4 | 7.69 |  |  |  |  |  |
| **68** | **CREBBP** | 7 | 13.46 | **143** | **FANCG** | 4 | 7.69 |  |  |  |  |  |
| **69** | **FGFR4** | 1 | 1.92 | **144** | **EP300** | 2 | 3.85 |  |  |  |  |  |
| **70** | **AXIN1** | 2 | 3.85 | **145** | **CDKN1A** | 3 | 5.77 |  |  |  |  |  |
| **71** | **FAM123B** | 6 | 11.54 | **146** | **TYRO3** | 2 | 3.85 |  |  |  |  |  |
| **72** | **ERCC4** | 2 | 3.85 | **147** | **MEN1** | 1 | 1.92 |  |  |  |  |  |
| **73** | **IRS2** | 6 | 11.54 | **148** | **SRC** | 9 | 17.31 |  |  |  |  |  |
| **74** | **BRCA1** | 3 | 5.77 | **149** | **TET2** | 7 | 13.46 |  |  |  |  |  |
| **75** | **KDR** | 4 | 7.69 | **150** | **MSH6** | 3 | 5.77 |  |  |  |  |  |
